# Supplementary material for: Magnetic Carbon Quantum Dots/Iron Oxide Composite Based on Waste Rice Noodle and Iron Oxide Scale: Preparation and Photocatalytic Capability
Source: Nanomaterials (Basel). 2023 Sep 6;13(18):2506. doi: 10.3390/nano13182506 (PMC10536646; doi:10.3390/nano13182506)
Supplement: Supplementary file 1 [file nanomaterials-13-02506-s001.zip › nanomaterials-2557429-SI.pdf]

nanomaterials

Supplementary Information

Magnetic carbon quantum dots/iron oxide composite based on waste rice noodle and iron oxide scale: preparation and photo-catalytic capability

Wanying Ying, Qing Liu, Xinyan Jin, Guanzhi Ding, Mengyu Liu, Pengyu Wang and Shuoping Chen*

[1] College of Materials Science and Engineering, Guilin University of technology, Guilin 541004, China; 2120210327@glut.edu.cn (W.Y.); 2120220336@glut.edu.cn (Q.L.); 2120200261@glut.edu.cn (X.J.); 1020220195@glut.edu.cn (G.J.); 2120210291@glut.edu.cn (M.L.); 2120220368@glut.edu.cn (P.W);

* Correspondence: 2012014@glut.edu.cn (S.C.);

S1 General characterization

The characterization of the CQDs/FeO_x composite material was performed with reference to similar characterization methods as outlined in our prior reports[1,2]. The powder X-ray diffraction (PXRD) patterns of CQDs/FeO_x composite and other control samples were acquired via an X'pert PRO X-ray diffractometer (Panalytical, Malvern, Worcestershire, UK) employing Cu Kα radiation (λ = 0.15418 Å) at 40 kV and 40 mA, with a scanning speed set at 5° per minute (2θ). Particle size distribution was determined using a Nano ZS type nanoparticle size and zeta point analyzer (Malvern, Malvern, UK) using a 633 nm laser. The morphology and EDS elemental analysis underwent characterization using a JEM-2100F field emission transmission electron microscope (TEM) from JEOL (Akishima, Tokyo, Japan) at an accelerating voltage of 200 kV. X-ray photoelectron spectroscopy (XPS) for both the CQDs/FeO_x composite and FeO_x was executed using an ESCALAB 250Xi X-ray photoelectron spectrometer (Thermo Fisher, Waltham, MA, USA), powered by an Al Kα X-ray as the excitation source. The obtained infrared (IR) spectra of the resultant products were recorded as KBr pellets over the range of 400 to 4000 cm^{-1}, utilizing a Nicolet 5700 FT-IR spectrometer (Thermo Fisher, Waltham, MA, USA) featuring a spectral resolution of 4.00 cm^{-1}. The determination of BET-specific surface areas for both the CQDs/FeO_x composite and FeOx was executed using a TriStar II 3020 surface area analyzer (Micromeritics, Atlanta, GA, USA) in a nitrogen environment. The assessment was conducted with an initial temperature set at 150 °C and a heating rate of 10 °C per second. To ascertain the points of zero charge (PZC) for the obtained samples, the following method was employed[1,2]: A sealed centrifugal tube containing 10 mL of 0.01 mol L^{-1} NaCl solution was employed. The pH of the solution was manipulated to fall between 2 and 11 by incremental additions of HCl or NaOH solutions. Subsequently, 0.05 g of the sample was introduced, and the final pH was gauged after 10 hours of agitation. The PZC was determined at the intersection of the pH final versus pH initial curve for the test sample and the control sample.

The UV-VIS absorption spectra of the result materials were meticulously characterized through employment of a UV3100 UV-VIS-NIR spectrophotometer (Shimadzu, Chiyoda, Tokyo, Japan) operating in the mode of diffuse reflection. For reference, $BaSO_4$ was employed as the benchmark material. Investigation into the photo-luminescence spectra of both the CQDs/FeO_x composite and FeO_x was conducted using a Cary Eclipse fluorescence spectrophotometer (Varian, Palo Alto, CA, USA), which employed a xenon lamp as its excitation source. The range spanned from 350 to 650 nm, with an excitation wavelength of 325 nm being applied. Evaluation of the photocurrent response (PCR) and electrochemical impedance spectra (EIS) was achieved through utilization of a CH1690 electrochemical analyzer (Huayan, Beijing, China). This apparatus was operated with a 0.5 V bias voltage while subjecting it to illumination from a 300 W Xe lamp, which acted as the source of light.

We delved into the effects of dynamic species like the superoxide radical (O_2^{-}), hydroxyl radical (·OH), and photogenerated hole (h+) within the progression of the photocatalytic reaction. To unravel their roles, diverse scavengers were introduced. Precisely, 2-propanol (IPA), 1,4-benzoquinone (BQ, with a purity of 98%), and ethylenediaminetetraacetic acid disodium salt (EDTA–2Na) were

strategically employed to pinpoint ·OH, O_2^{-}-, and h+, respectively. Employing a methodology akin to the prior photocatalytic activity assay[1,2], we incorporated 1 mmol of the scavenger into the reaction mixture. In tandem, electron spin resonance (ESR) spectroscopy was harnessed to gauge the proficiency of radical generation within the CQDs/FeO_x composite and FeO_x under 405 nm visible light irradiation. For this purpose, we employed an EMXplus X-band electron paramagnetic resonance spectrometer (Bruker, Karlsruhe, Baden-Württemberg, Germany), with dimethyl pyridine N-oxide (DMPO) serving as the spin-trapping agent for ESR measurements.

S2 Measurement of photocatalytic performance

Similar to the method employed in our previous work[1,2], the examination of photocatalytic degradation experiments involving organic pollutants was carried out under the influence of visible light irradiation. To commence, a quantity of 2 g/L of the CQDs/FeO_x composite was uniformly dispersed within an aqueous solution containing organic pollutants at an initial concentration of 20 mg/L. This amalgamation underwent shaking on an OS-20 shaker table (Oaiclab, Zhejiang, China), maintained at 25 °C in darkness, for a duration of 30 minutes. Subsequently, the mixture was positioned beneath a 20 W 405 nm purple light lamp emitting light at an intensity of 8×10^4 LUX. This purple light lamp emitted wavelengths ranging from 340 to 450 nm (Refer to Figure S7 in ESI). Repetitive experiments were meticulously prepared as individual samples. At designated time intervals, one of the samples was extracted and subsequently subjected to centrifugation. The concentrations of organic pollutants within these samples were quantified using UV-VIS spectrophotometry through utilization of a UV3100 UV-VIS-NIR spectrophotometer (Shimadzu, Chiyoda, Tokyo, Japan). The testing wavelengths employed were 664 nm (pertaining to methylene blue) and 357 nm (relating to tetracycline hydrochloride). The extent of photocatalytic degradation pertaining to the organic pollutants was determined by evaluating the C/C_0 value, where C represents the concentration of the organic pollutant at a specific time and C_0 symbolizes the initial concentration of the organic pollutant. It's important to note that each outcome of photocatalytic degradation reported in this study reflects the average measurement derived from three samples sharing identical compositions.

The photocatalytic kinetics can be elucidated through the Langmuir-Hinshelwood model, wherein the integral representation is as follows:

$$t = (\frac{1}{K_r K})\ln(\frac{C_0}{C}) + \frac{C_0 - C}{K_r} \quad (1)$$

Here, *t* signifies the irradiation time; *K* stands for the equilibrium constant governing the adsorption of the organic pollutant on the catalyst, while K_r reflects the reaction's limiting rate at the utmost coverage within the given circumstances; C_0 denotes the initial concentration of the organic pollutant, and *C* represents the pollutant concentration at time t.

When the organic pollutant's initial concentration is low, the second term in equation (1) becomes negligible, and therefore, it can be disregarded. Consequently, the kinetics analysis for photocatalytic degradation in this study employed the subsequent equation:

$$\ln(\frac{C_0}{C}) = K_r K t = K_{app} t \quad (2)$$

Here, K_{app} stands for the apparent degradation rate constant, measured in min^{-1}, serving as the fundamental kinetic parameter.

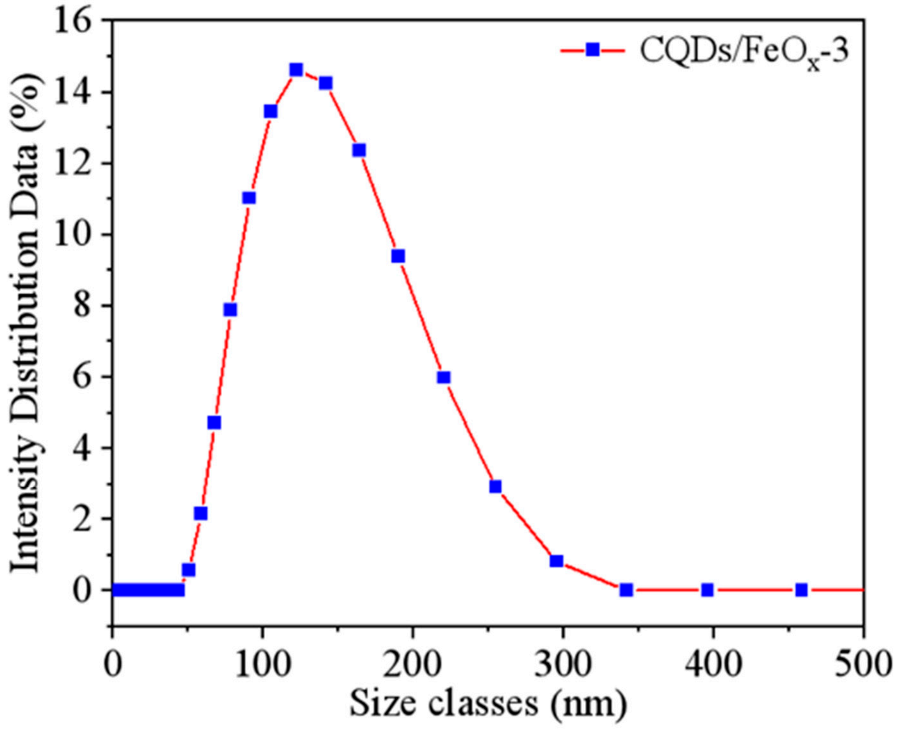

Figure S1 The particle size distribution of CQDs/FeO_x–3 sample.

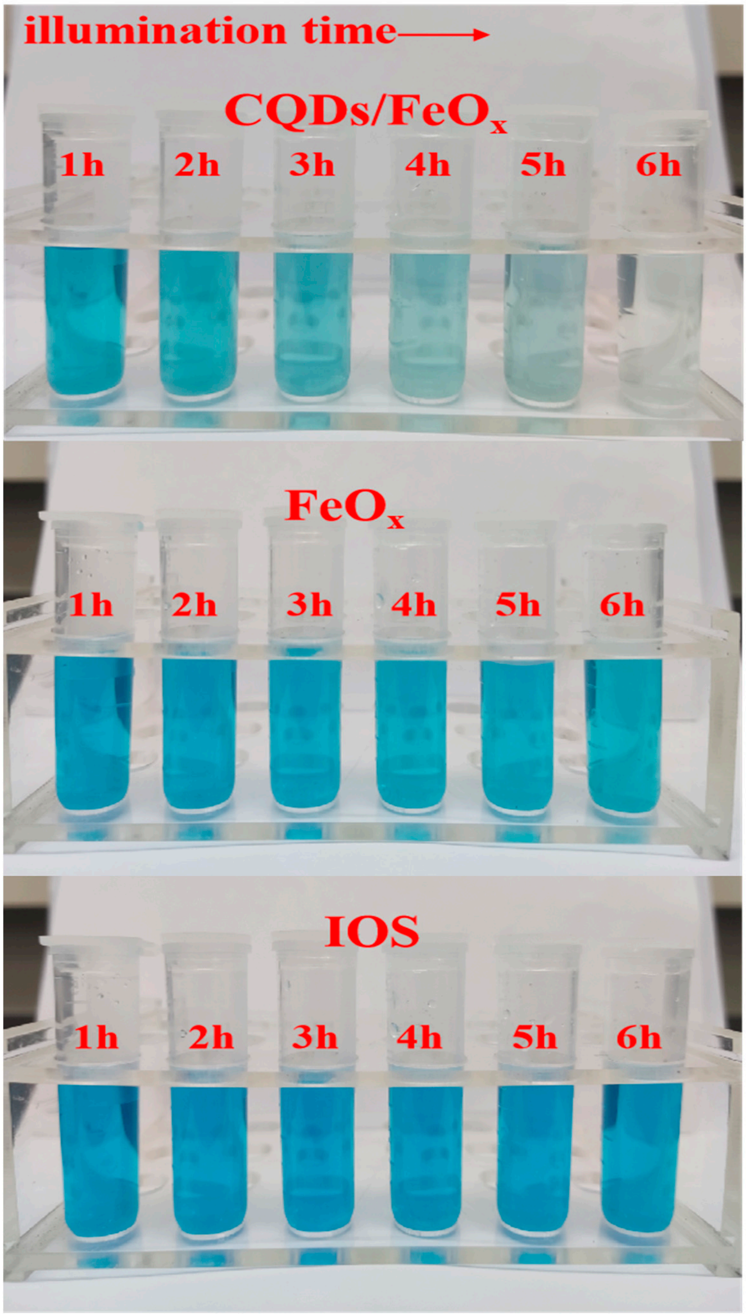

Figure S2 Comparative images of photocatalytic degradation of methylene blue by CQDs/FeO_x composites, IOS and FeO_x.

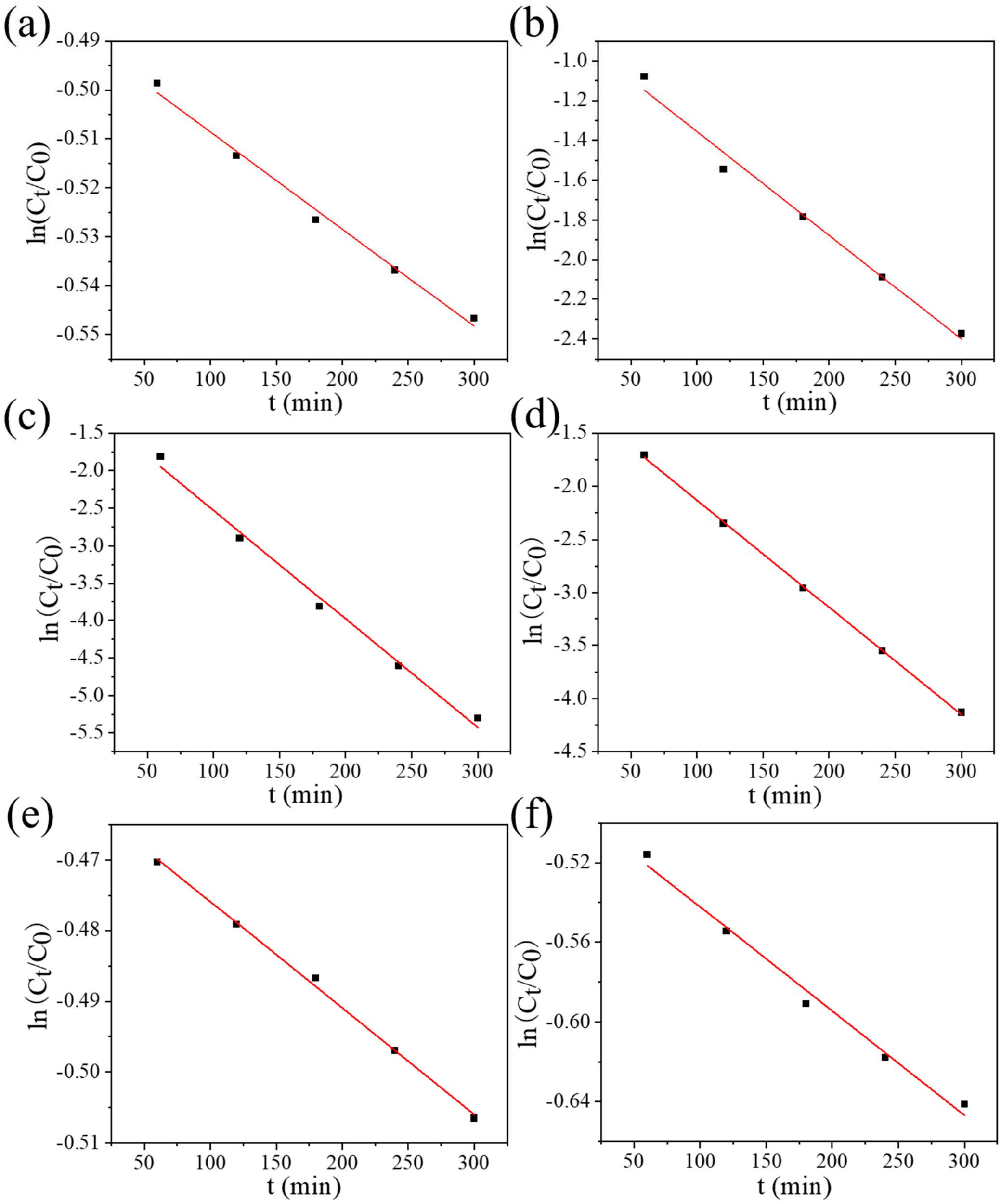

Figure S3 The pseudo-first-order kinetic fitting of photocatalytic degradation of CQDs/FeO_x–1 sample (a), CQDs/FeO_x–2 sample (b), CQDs/FeO_x–3 sample (c), CQDs/FeO_x–4 sample (d), CQDs/FeO_x–5 sample (e) and FeO_x (f) to methylene blue within different irradiation times under 405 nm purple light.

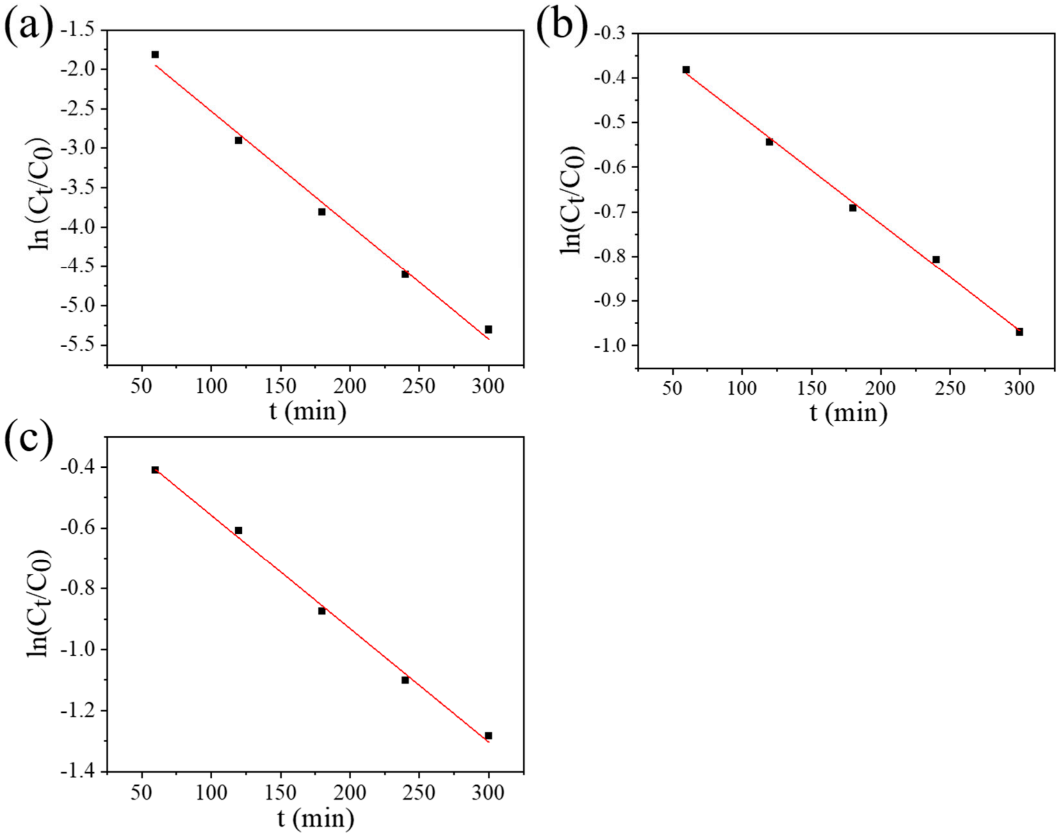

Figure S4 The pseudo-first-order kinetic fitting of photocatalytic degradation of CQDs/FeO_x–3 sample (a), CQDs/FeO_x–6 sample (b), CQDs/FeO_x–7 sample (c) to methylene blue within different irradiation times under 405 nm purple light.

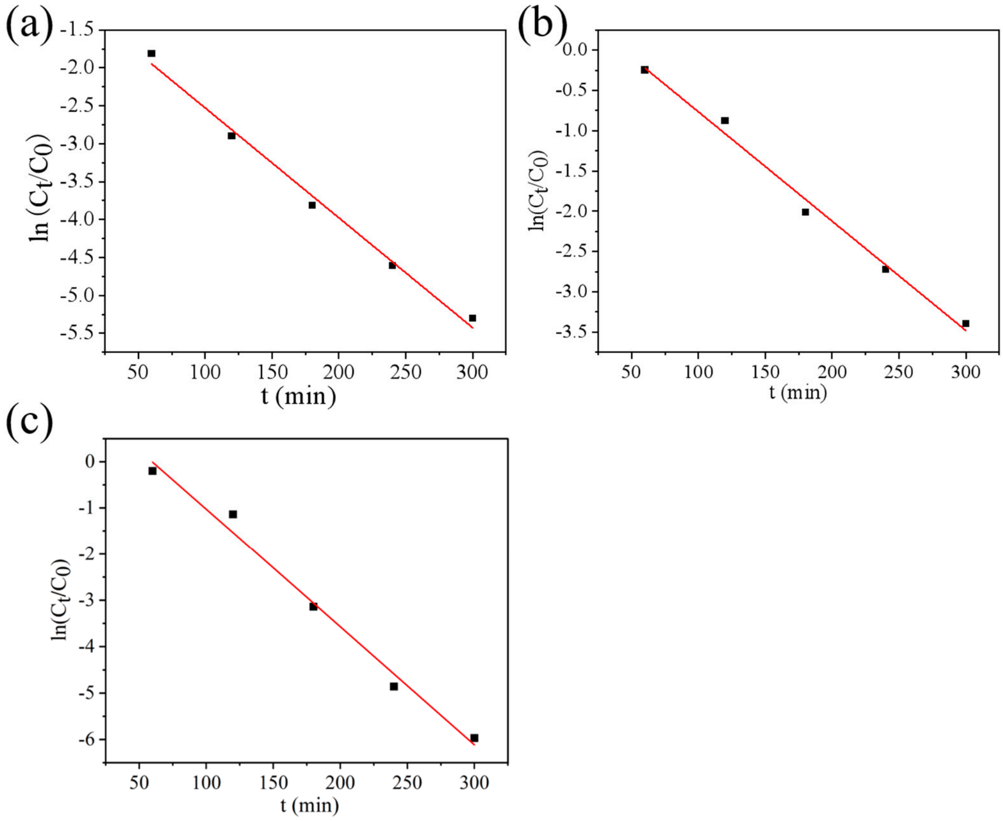

Figure S5 The pseudo-first-order kinetic fitting of photocatalytic degradation of CQDs/FeO_x−3 sample (a), TiO_2(b), ZnO(c) to methylene blue within different irradiation times under 405 nm purple light.

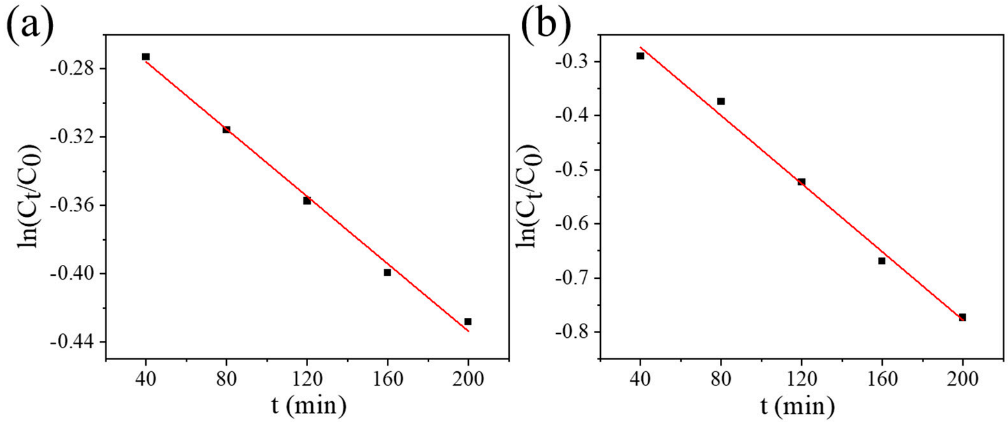

Figure S6 The pseudo-first-order kinetic fitting of photocatalytic degradation of FeO_x (a) and CQDs/FeO_x–3 sample (b) to tetracycline hydrochloride within different irradiation times under 405 nm purple light.

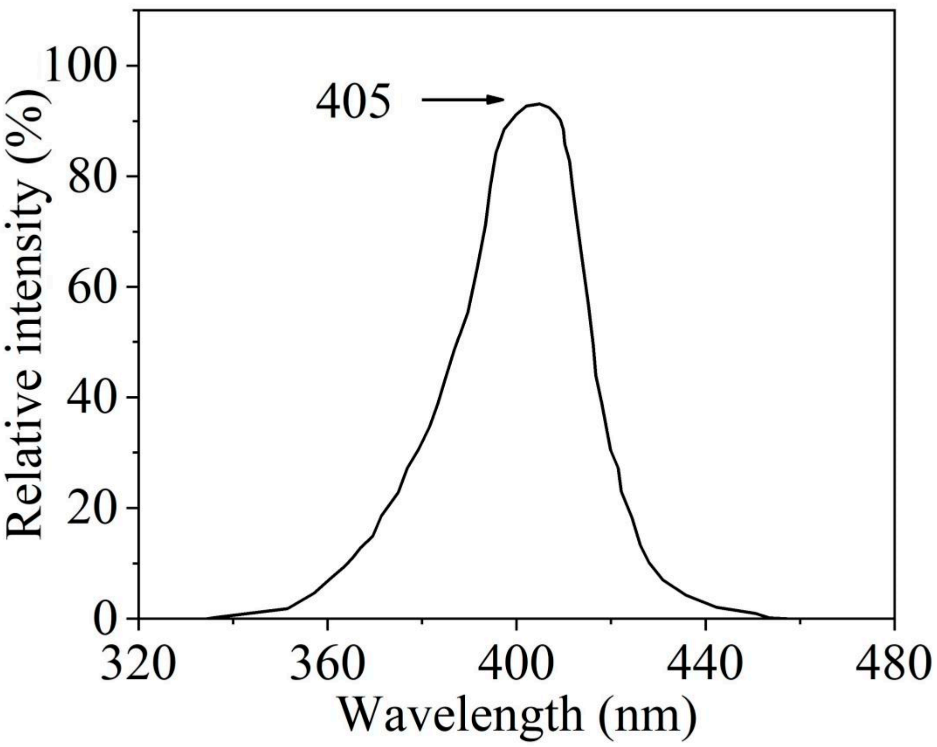

Figure S7 The emission spectrum of the 405 nm purple light lamp.

Table S1 The kinetic parameters of photocatalytic degradation to methylene blue of CQDs/FeO_x composites, FeO_x, commercial TiO_2 and ZnO under 405 nm purple light.

Photocatalyst	K_{app} ($\times10^{-3}min^{-1}$)	R^2
CQDs/FeO_x-1	0.70	0.97537
CQDs/FeO_x-2	2.02	0.99733
CQDs/FeO_x-3	5.26	0.99905
CQDs/FeO_x-4	3.89	0.99501
CQDs/FeO_x-5	1.32	0.99832
CQDs/FeO_x-6	2.40	0.99729
CQDs/FeO_x-7	3.73	0.99650
FeO_x	1.02	0.99038
Commercial TiO_2	13.57	0.99030
Commercial ZnO	25.44	0.98754

Table S2 The kinetic parameters of photocatalytic degradation to tetracycline hydrochloride of CQDs/FeO_x-3 sample and FeO_x and under 405 nm purple light.

Photocatalyst	K_{app} ($\times10^{-3}min^{-1}$)	R^2
CQDs/FeO_x-3	3.15	0.99215
FeO_x	0.99	0.99541

References

1. Jin, X.; Che, R.; Yang, J.; Liu, Y.; Chen, X.; Jiang, Y.; Liang, J.; Chen, S.; Su, H. Activated Carbon and Carbon Quantum Dots/Titanium Dioxide Composite Based on Waste Rice Noodles: Simultaneous Synthesis and Application in Water Pollution Control. *Nanomaterials.* **2022**, *12*, 472.
2. Jin, X.Y.; Ying, W.Y.; Che, R.J.; Xiao, P.; Zhou, Y.Q.; Liu, Y.; Liu, M.Y.; Chen, S.P. CQDs/ZnO composites based on waste rice noodles: preparation and photocatalytic capability†. *RSC Advances.* **2022**, *12*, 23692-23703.
